# Professor Richard Von Volkmann

**Published:** 1890-01

**Authors:** 


### Obituary.

PROFESSOR RICHARD VON VOLKMANN died at Halle, November 28th. He was born in Leipsic August 17, 1830, began the study of medicine in Halle in 1850. In 1857 he was made assistant in surgery under Blasius, after whose death in 1867 he was made Professor of Surgery and Director of the Surgical Clinic, which positions he held at the time of his death.

He was the first to practise antisepsis in Germany, and won, through his admirable technique and clear teaching, the reputation of being Germany's ablest surgeon. No one did so much as he to throw light upon bone diseases, and especially the tuberculous forms of it. He was made a nobleman in 1885 in recognition of his surgical ability.

